# Lead levels in fur of rats treated with inorganic lead measured by inductively coupled argon plasma mass spectrometry

**DOI:** 10.2478/v10102-010-0050-y

**Published:** 2010-12

**Authors:** François-Xavier Lesage, Frédèric Deschamps, Hervé Millart

**Affiliations:** 1 Department of Occupational Health, Institut de Médecine du Travail, Faculté de Médecine, Reims, France; 2 Laboratory of Pharmacology and Toxicology, Centre Hospitalier Régional et Universitaire, Reims, France

**Keywords:** fur lead, hair lead, spectrometry, rat

## Abstract

The aim of this study was to investigate the relationship between continuous lead exposure and the concentration of this metal in fur. The two main questions we wanted to answer were: 1) Are the fur lead concentrations different according to exposure level? 2) Is the kinetics of lead concentration linear in different compartments?

For 12 weeks, 6 rats were force-fed with water containing lead acetate in the following quantities: 0.5 and 50 µg/day. Furs were sampled every two weeks. The lead content of the samples was measured by inductively coupled argon plasma mass spectrometry (ICP-MS).

There was a statistical difference (*p<*0.0001) between fur lead concentration and the three groups (control, low level exposure and high level exposure), between fur lead concentration and time exposure (*p<*0.0001), and between fur lead concentration and each exposure group at different time exposure (*p<*0.0001). Thus the level exposure factor and the time exposure factor have an effect on fur lead concentration. Since the determination coefficients were weak for the two exposed groups (0.032 and 0.032), a linear correlation cannot be concluded. The kinetic curves of fur lead concentration are similar for all the exposition groups. Two peaks (at 2 and 8 weeks of exposure) were noted for the two exposed groups.

This experimental study cannot conclude a linear relationship to exist between fur lead concentration and exposition duration. It highlights the lack of understanding of mechanisms involved in hair incorporation of metals and raises the question of a cyclic accumulation in hair. A better understanding of the kinetic incorporation of lead in body growths is required.

## Introduction

Blood lead (Pb) concentration is at the present the best biological marker of lead exposure over the preceding weeks when exposure is stable. It increases from the first exposure day to the third month, and steady-state occurs at the third month. It returns quickly to baseline level after one month without exposure (Rabinowitz *et al*., 1976a). So, some weeks after an irregular exposure, the body pool of lead may be underestimated because the blood lead concentration may have already decreased. Inversely, this pool may be overestimated after just an acute exposure. Blood lead concentration is correlated to constant and recent exposure, for at least the following three months.

Trace element analysis on hair samples has been widely used to assess wildlife and human exposure to different contaminants present in the environment or in the workplace (Pereira *et al*., 2004).

There are several reported advantages of using this biological material for monitoring (ATSDR, 2005): (i) the less invasive nature of hair collection procedures; (ii) the stability of hair and nails as a biological material (Deschamps *et al*., 2004), which facilitates sample storage and transportation; (iii) the higher concentrations of residues usually found in hair samples, as compared to those in blood and urine; and (iv) the capacity of hair to accumulate metals during extended periods, reflecting by this way at least one year of exposure. Nevertheless, hair analysis has limitations, mainly due to the occurrence of exogenous contamination (ATSDR, 2005). The main sources of exogenous contamination are deposit of sebum, sweat, polluted air residues, and residues of cosmetic or pharmaceutical products.

Other shortcomings of hair analysis include: (i) the lack of correlation between the concentrations of trace elements in hair and in the other target organs (*e.g.* liver, kidney) or body fluids (*e.g.* blood, urine); (ii) the poorly understood kinetics of incorporation of trace elements in hair; and (iii) the lack of epidemiological data to predict health effects resulting from the presence of a trace element in hair. Despite these limitations in order to assess quantitatively the exposure, scalp hair has been considered a good sampling site in order to screen for possible environmental exposures of a population that would justify more extensive studies.

Actually, for most substances, the data are insufficient to predict health effects from the concentration of the substance in hair. Before hair analysis can be considered a valid tool for assessing quantitatively the exposure and its health impact, research is needed to establish standardized reference ranges, to gain a better understanding of biologic variations of hair growth with age, gender, race and ethnicity, and pharmacokinetics, and further explore possible dose-response relationships (Harkins & Susten, 2003).

There are a number of studies relating lead exposure to tissue concentrations, including hair.. During the exposure period, one animal study showed a dose-dependent correlated increase of lead in bone and hair (Hac & Krechniak, 1996). Isotopic tracer studies have shown cumulative pooling over three months of lead into human facial hair (Rabinowitz *et al*., 1976b).

In humans, hair analysis can be used to demonstrate lead poisoning (Watt et al. 1990). In occupational exposures there is a correlation between blood and hair lead concentrations (Foo *et al*., 1993; Taylor, 1986). Such an association has also been noted with low-level exposure, but only in the larger studies (Taylor, 1986; Wilhelm & Idel, 1996).

The Agency for Toxic Substances and Disease Registry (ATSDR) encourages researchers to continue in the development of valid analytic techniques that can accurately measure specific hazardous substances in human hair. Applied research is needed to test new technology, establish reference ranges, understand pharmacokinetics, and explore time and dose relationships.

The aim of this study was to investigate the potential correlation between some low level lead exposures and their rates measured in fur of rats. The two main questions we wanted to answer were:

1. Are the fur lead concentrations different according to exposure level?
2. Is the kinetics of lead concentration linear in different compartments?

## Material and methods

### Type of study

Experimental survey

### Duration of study

Three months

### Animal studies

Eighteen 7-week-old male Sprague-Dawley rats were used, caged in groups of three animals. Two cages were randomly assigned to each experimental group.

### Exposition

The control group was exposed to dietary lead. The lead concentration in their tap water had been previously measured. It was below 0.5 µg/l.

For twelve weeks, rats included in the two exposed groups were tube-fed 1 ml of water containing lead acetate (C_4_H_12_Pb.3H_2_O) in different concentrations to deliver either 0.5 µg/day of lead (low level group) or 50 µg/day (high level group). Dietary lead exposure ceased after 12 weeks.

### Sample collection

Animal hair was collected with shears from a 1 cm^2^ area on the back, using the same location for all the animals. Each sample was weighed then mineralized. The sampling was done for each rat on day 0, then every 14 days for 12 weeks. The cages were randomly assigned to the different treatment groups.

### Lead analytical method

The hair samples were acid mineralized (1 ml H_2_O_2_ and 3 ml HNO_3_ (RP 65% Merck^®^) and diluted with HNO_3_ (1%) to make up to 25 ml. A Hewlett Packard^®^ HP 4500 Mass 208 inductively coupled argon plasma mass spectrometer (ICP M-S) was used to analyze the different samples. Its detection threshold was 0.046 µg/l. Analyses were done in triplicate.

### Data analysis

We used the software StatView^®^ for Windows v. 5.0 (SAS Institute Inc.) for our statistical analyses. The Pb concentrations in hair (dependent variable) were statistically analyzed for differences following the 0.5 µg/dayand the 50 µg/d doses. Means, confidence intervals and statistical significances were evaluated with Student t-test. Kolmogorov-Smirnov test was used to test the normal hypothesis of the dependent variable. Longitudinal data analysis was tested with “repeated measures” analysis of variance (ANOVA). The linearity hypothesis was tested by using regression analysis to determine the coefficient of determination R^2^

## Results

The lead concentration in tap water is weak (0.5 µg/l).

One rat in the control group and one in the high exposure level group died during the study. Their fur lead concentrations were excluded from the statistical analysis. The mean values of fur lead concentrations are statistically significant (Table 1). Two peaks of fur lead concentration are noted, at 2 and 8 weeks in all the groups studied.

**Table 1:** Concentration of lead in hair of rats treated with lead acetate.

	Initial concentration	2 weeks	4 weeks	6 weeks	8 weeks	10 weeks	12 weeks
Control	0.4±0.28*	7.16±5.2	0.6±0.26	1.81±0.92	2.33±1.37	1.29±1.26	1.51±1.12
n=5	*p=*0.017	*p=*0.019	*p=*0.003	*p=*0.005	*p=*0.009	*p=*0.046	*p=*0.02
Low Level group	0.65±0.5	4.81±1.2	2.07±0.79	1.73±0.37	5.49±1.69	4.05±1.13	2.58±0.98
n=6	*p=*0.021	*p*<0.001	0.001	*p*<0.001	*p*<0.001	*p*<0.001	*p*<0.001
High level group	0.02±0.05	43.89±15.28	23.6±8.96	15.5±8.96	69.16±23.61	34.63±19.35	36.71±31.7
n=5	*p=*0.374	*p=*0.001	*p=*0.0018	*p=*0.008	*p=*0.001	*p=*0.0076	*p=*0.009

Concentration ± confidence interval

### “Repeated measures” ANOVA

There is a statistical difference (*p<*0.0001) between fur lead concentration and the three groups, between fur lead concentration and time exposure (*p<*0.0001), and between fur lead concentration and each exposure group at different times of exposure (*p<*0.0001) (Table 2). The lead concentrations are different according to exposure level and time exposure.

**Table 2:** “Repeated Measures” Analysis of variance of fur lead concentration in terms of group factor and time exposure factor.

Factors	*p-*value
Group	<0.0001
Time exposure	<0.0001
Group*time exposure	<0.0001

### Regression analysis

The coefficients of determination R^2^ between fur lead concentration and time are weak for the two groups of different exposure level, 0.032 and 0.175, respectively (Figures 2 and 3). Thus a linear correlation cannot be concluded.

## Discussion

The choice of a “repeated measures” design study was led with respect to the three R rule: Replace, Reduce, Refine. This design allows to reduce the number of rats, but it makes more complex the analysis. The same animals are repeatedly tested at different times. Consequently, the outcome variable is not independent across time. We chose to process these longitudinal data with a three-way analysis of variance, time being a “within” factor, and lead level exposure a “between” factor. Moreover, to “Reduce and Refine”, we found it relevant to compare fur lead concentration direct to the lead intake rather than using another biomarker such as bone lead concentration. The design imposes a control of lead intakes by tube-feeding rather than by tap water *ad libitum*.

The advantages of the ICP-MS applied to hair analysis is its high sensitivity, with a detection threshold of 0.046 µg/l (inferior to 0.07 mg/g of hair), and its specificity, which allow a quick analysis of different metals on a single hair sample (Miekeley *et al*., 1998).

The ICP-MS seems to be able to discriminate the different exposurelevels (Tables 1 and 2). There are many authors who plead in favour of this technique for the determination of lead, and probably some other metals in superficial body growths (Miekeley *et al*., 1998; Goulle *et al*., 2005).

**Figure 1:**
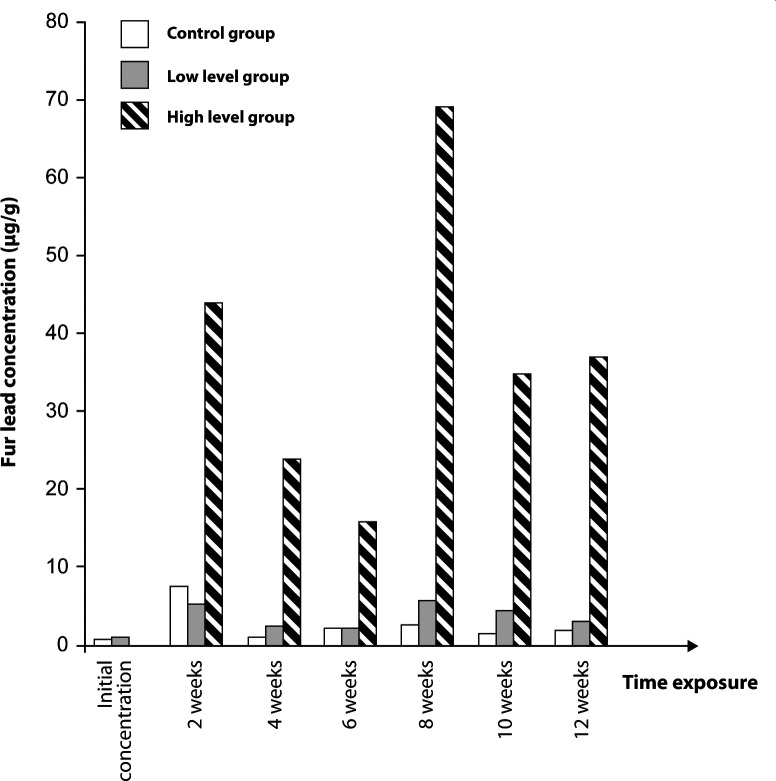
Lead content in fur of rats treated with lead acetate

**Figure 2:**
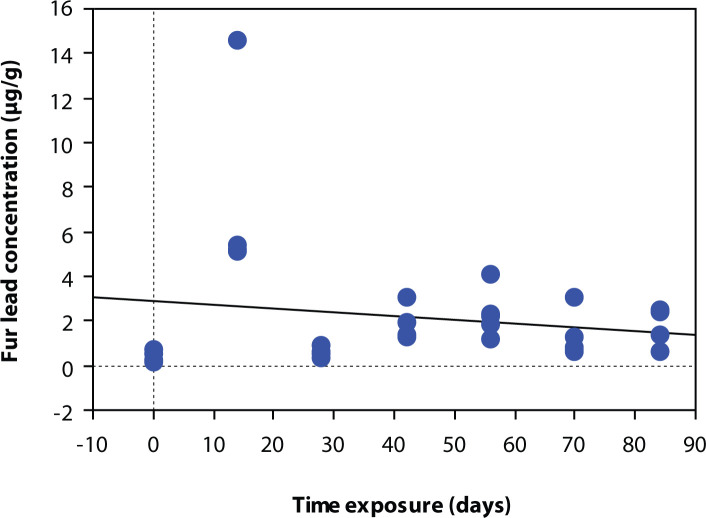
Relationship between fur lead concentration (µg lead/g hair) and time exposure (days) for low level (0.5 µ/day) lead exposure. Regression equation: Fur lead concentration (µg/g) = 2.868 – 0.017 × time exposure (days). Coefficient of determination: R^2^=0.032

We found a relationship between fur lead concentration and the different exposure levels and exposure duration.. But, in contrast to what Hac and Krechniak (1996) reported, we were unable to demonstrate a linear relationship between lead concentration and duration of exposition. (Figures 1 and 2). However, the experiment designs were different.

**Figure 3:**
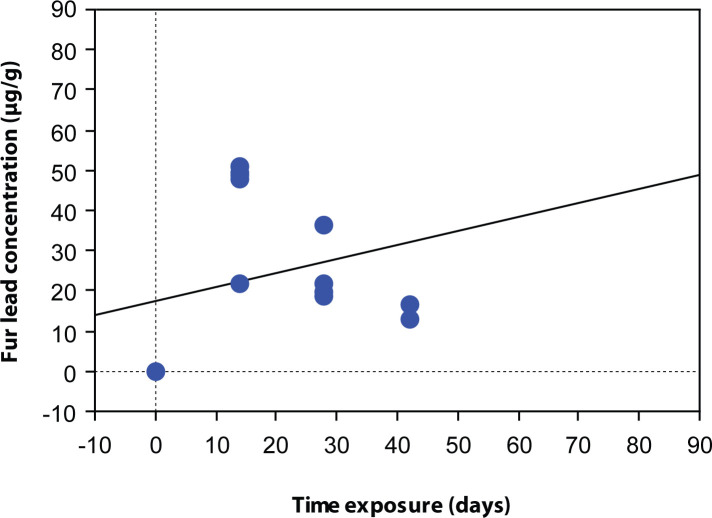
Relationship between fur lead concentration (µg/g) and time exposure (days) for high level (50 µ/day) lead exposure. Regression equation: Fur lead concentration (µg/g) = 17.23 + 0.35 × time exposure (days). Coefficient of determination: R^2^=0.175.

The kinetics of fur lead is non-linear in our results, but similar for the two exposition groups, with a peak at 2 and 8 weeks. This result could be due to an external contamination, but the similarity for the two groups raises the question of a cyclic accumulation in hair. The results have to be confirmed by further studies.

Metal hair incorporation kinetics can be best understood by an isotopic study of lead bioavailability in hair by ICP-MS, allowing a better understanding of the mechanisms involved.

Some bias must be taken into account. Some variations of growth of fur (this study continued for three months) could interfere with the results. They may include variations of lead absorption according the rats’ age (they were seven weeks old at the beginning of the exposure) and individual variability. This should be studed by some other exposure pathways.

The contamination of samples must be taken into account. Even if there was a weak dispersion for the lead concentration in every group sample, the contamination of a sample is the main and recurrent problem in hair analysis.

## Conclusion

This experimental study pleads in favour of a relationship between exposure level, time exposure, and fur lead concentration, but cannot conclude a linear relationship between fur lead concentration and exposition duration. It highlights the lack of understanding of mechanisms involved in hair incorporation of metals and raises the question of a cyclic accumulation in hair, with more complex incorporation mechanisms than a linear relationship would suggest.

## References

[CIT0001] ATSDR Hair analysis panel discussion: exploring the state of the science. [webpage on the Internet]. Summary report. Atlanta. GA: Agency for Toxic Substances and Disease Registry.

[CIT0002] Deschamps F, Fouley A, Habets F, Arsac F, Cser MA, Sziklay IL, Etienne JC, Raymar I, Centeno J, Kassanova L, Collery PH (2004). Relationships between nails lead level and usual markers in occupational workers. Metal ions in biology and medicine.

[CIT0003] Foo SC, Khoo NY, Heng A, Chua LH, Chia SE, Ong CN, Ngim CH, Jeyaratnam J (1993). Jeyaratnam. Metals in hair as biological indices for exposure. Int Arch Occup Environ Hlth.

[CIT0004] Goullé JP, Mahieu L, Castermant J, Neveu N, Bonneau L, Lainé G, Bouige D, Lacroix C (2005). Lacroix. Metal and metalloid multi-elementary ICP-MS validation in whole blood, plasma, urine and hair. Reference Values. Forensic Sci Int.

[CIT0005] Harkins DK, Susten AS (2003). Hair analysis: exploring the state of the science. Environ Hlth Persp.

[CIT0006] Hac E, Krechniak J (1996). Lead levels in bone and hair of rats treated with lead acetate. Biol Tr Elem Res.

[CIT0007] Miekeley N, Dias Carneiro MT, da Silveira CL (1998). How reliable are human hair reference intervals for trace elements. Sci Total Environ.

[CIT0008] Pereira R, Ribeiro R, Gonçalves F (2004). Scalp hair analysis as a tool in assessing human exposure to heavy metals (S. Domingos mine, Portugal). Sci Total Environ.

[CIT0009] Rabinowitz MB, Wetherhill GW, Kopple JD (1976a). Kinetic analysis of lead metabolism in healthy humans. J Clin Invest.

[CIT0010] Rabinowitz MB, Wetherill GW, Kopple JD (1976b). Delayed appearance of tracer lead in facial hair. Arch Environ Health.

[CIT0011] Taylor A (1986). Usefulness of measurements of trace elements in hair. Ann Clin Biochem.

[CIT0012] Watt F, Landsberg J, Powell JJ, Ede RJ, Thompson RP, Cargnello JA (1990). Analysis of copper and lead in hair using the nuclear microscope; results from normal subjects and patients with Wilson's disease and lead poisoning. Analyst.

[CIT0013] Wilhelm M, Idel H (1996). Hair analysis in environmental medicine. Zbl Hyg.

